# A RESTful application programming interface for the PubMLST molecular typing and genome databases

**DOI:** 10.1093/database/bax060

**Published:** 2017-08-09

**Authors:** Keith A. Jolley, James E. Bray, Martin C. J. Maiden

**Affiliations:** 1 Department of Zoology, Peter Medawar Building for Pathogen Research, University of Oxford, South Parks Road, Oxford, OX1 3SY, UK

## Abstract

Molecular typing is used to differentiate microorganisms at the subspecies or strain level for epidemiological investigations, infection control, public health and environmental sampling. DNA sequence-based typing methods require authoritative databases that link sequence variants to nomenclature in order to facilitate communication and comparison of identified types in national or global settings. The PubMLST website (https://pubmlst.org/) fulfils this role for over a hundred microorganisms for which it hosts curated molecular sequence typing data, providing sequence and allelic profile definitions for multi-locus sequence typing (MLST) and single-gene typing approaches. In recent years, these have expanded to cover the whole genome with schemes such as core genome MLST (cgMLST) and whole genome MLST (wgMLST) which catalogue the allelic diversity found in hundreds to thousands of genes. These approaches provide a common nomenclature for high-resolution strain characterization and comparison. Molecular typing information is linked to isolate provenance, phenotype, and increasingly genome assemblies, providing a resource for outbreak investigation and research in to population structure, gene association, global epidemiology and vaccine coverage. A Representational State Transfer (REST) Application Programming Interface (API) has been developed for the PubMLST website to make these large quantities of structured molecular typing and whole genome sequence data available for programmatic access by any third party application. The API is an integral component of the Bacterial Isolate Genome Sequence Database (BIGSdb) platform that is used to host PubMLST resources, and exposes all public data within the site. In addition to data browsing, searching and download, the API supports authentication and submission of new data to curator queues.

**Database URL:** http://rest.pubmlst.org/

## Introduction

The PubMLST website (https://pubmlst.org) was established in 2003 to host multi-locus sequence typing (MLST) data, initially for the *Neisseria* (1) and *Campylobacter* (2) schemes developed at the University of Oxford, although its origins date back to the first MLST site established in 1998 (http://mlst.zoo.ox.ac.uk) (1). Conventional MLST schemes consist of collections of housekeeping gene fragment loci, usually seven each about 500 bp in length, for which (i) every new allelic variant is assigned an integer identifier; and (ii) every unique profile consisting of the combination of these alleles a sequence type (ST) number (1). This provides a concise summary of the variation across these loci, data compression, a simple nomenclature, and a rapid means of comparison by counting the number of loci that differ between any pair of isolates (3). For example, in *Neisseria*, ST-11 represents the unique combination of alleles of *abcZ*:2, *adk*:3, *aroE*:4, *fumC*:3, *gdh*:8, *pdhC*:4, *pgm*:6. A single nucleotide variation at any of these loci defines a different allele, and consequently a different ST, so all isolates with a particular ST share the exact same sequence across these loci. Related isolates will share alleles at some of the loci and STs can be grouped in to clonal complexes based on the numbers of shared alleles. Since its inception, PubMLST has offered hosting for MLST and single-gene schemes from the community and there are now schemes for over a hundred, mainly bacterial, microorganisms on the site. As a collection of curated databases, PubMLST receives thousands of submissions a year from end users. These comprise: new sequence variants for allele assignment; new profiles consisting of combinations of alleles for ST assignment; and isolate records, with or without accompanying whole genome assemblies. The databases for each of the hosted organisms are curated by teams of domain specialists recruited internationally. Curators validate new sequence data and allelic profiles submitted by end users, ensuring that any new variant is real before being assigned an identifier and that this can be associated with representative isolate information.

In 2006, a Simple Object Access Protocol (SOAP) (https://www.w3.org/TR/soap12/) application programming interface (API) was introduced to provide programmatic access to MLST data to third party clients. This has been used extensively primarily by commercial software clients including BioNumerics (http://www.applied-maths.com/) and SmartGene (http://www.smartgene.com/) to synchronize typing definitions to be used within their applications. This interface was developed, however, to interact with the databases powered by an early implementation of the MLST software, mlstdbNet (4), and is limited to accessing and querying MLST data.

With the advent of routinely available whole genome sequence (WGS) data, the BIGSdb platform (5) was developed to host, flexibly organize, and extract allelic variants for any locus of interest: it was designed to be able to store both the genome sequence data as draft or complete assemblies, associated with its provenance metadata, along with any number and size of typing schemes and nomenclatures, ranging from the existing conventional MLST schemes through to collections of loci that make up the core-[e.g. core genome MLST, cgMLST (6–10)] and pan-genomes of a species or genus (11). These genome-level MLST schemes index allelic variation over hundreds or thousands of loci, representing the complete coding sequences of genes, but can be analysed in the same way as conventional MLST, by counting allelic differences among isolates (5, 6), which provides rapid high-resolution means of isolate discrimination. Many of the species-specific databases accept genome submissions and there are now over 55 000, mainly draft, genome assemblies in these databases, curated and linked to provenance, typing nomenclatures, and publications.

A Representational State Transfer (REST) (12) API has now been developed as an integral component of BIGSdb, facilitating the exposure of all public data held within the site from any programming environment. The API supports OAuth authentication so that users can delegate their access to third party tools to connect to protected resources or to submit data to the curation queues of databases supporting this feature. This API is accessible from http://rest.pubmlst.org/.

The source code for BIGSdb, including the RESTful API application, can be found at https://github.com/kjolley/BIGSdb. The API is fully documented at http://bigsdb.readthedocs.io/en/latest/rest.html.

## Implementation

The BIGSdb RESTful API is implemented as a Dancer2 (http://perldancer.org/) application, fully integrated in to the BIGSdb codebase (5), so that it utilizes the same library methods called by the web application code. It is run using the Starman high-performance pre-forking PSGI/Plack web server. The API receives queries via standard web calls utilizing HTTP verbs (GET, POST and DELETE) to signify the actions that should be taken (Table 1). Results are returned in JavaScript Object Notation (JSON) format, except for methods that specifically request bulk sequence data in FASTA format or profile data in tab-separated text files. Methods called with POST encode their parameters in JSON format within the payload of the call.

**Table 1.** PubMLST API methods*

**URI (to go after**http://rest.pubmlst.org**)**	HTTP method	Description
/	GET	List site resources
/db/{database}	GET	List database resources
/db/{database}/loci	GET	List loci
/db/{database}/loci/{locus}	GET	Retrieve locus record
/db/{database}/loci/{locus}/alleles	GET	Retrieve list of alleles defined for a locus
/db/{database}/loci/{locus}/alleles_fasta	GET	Download alleles in FASTA format
/db/{database}/loci/{locus}/alleles/{allele_id}	GET	Retrieve full allele information
/db/{database}/loci/{locus}/sequence	POST	Query sequence to identify allele
/db/{database}/sequence	POST	Query sequence to identify allele without specifying locus
/db/{database}/schemes	GET	List schemes
/db/{database}/schemes/{scheme_id}	GET	Retrieve scheme information
/db/{database}/schemes/{scheme_id}/fields/{field}	GET	Retrieve scheme field information
/db/{database}/schemes/{scheme_id}/profiles	GET	List allelic profiles defined for scheme
/db/{database}/schemes/{scheme_id}/profiles_csv	GET	Download allelic profiles in tab-delimited text format
/db/{database}/schemes/{scheme_id}/profiles/{profile_id}	GET	Retrieve allelic profile record
/db/{database}/isolates	GET	Retrieve list of isolate records
/db/{database}/isolates/{isolate_id}	GET	Retrieve isolate record
/db/{database}/isolates/{isolate_id}/allele_designations	GET	Retrieve list of allele designations
/db/{database}/isolates/{isolate_id}/allele_designations/{locus}	GET	Retrieve full allele designation record
/db/{database}/isolates/{isolate_id}/allele_ids	GET	Retrieve allele identifiers (abbreviated allele designations)
/db/{database}/isolates/{isolate_id}/schemes/{scheme_id}/allele_ designations	GET	Retrieve scheme allele designations records
/db/{database}/isolates/{isolate_id}/schemes/{scheme_id}/allele_ids	GET	Retrieve list of scheme allele identifiers
/db/{database}/isolates/{isolate_id}/contigs	GET	Retrieve list of sequence contigs
/db/{database}/isolates/{isolate_id}/contigs_fasta	GET	Download isolate contigs in FASTA format
/db/{database}/contigs/{contig_id}	GET	Retrieve contig record
/db/{database}/isolates/search	POST	Search isolate database
/db/{database}/fields	GET	Retrieve list of isolate provenance field descriptions
/db/{database}/fields/{field}	GET	Retrieve values set for a provenance field
/db/{database}/users/{user_id}	GET	Retrieve submitter/curator information
/db/{database}/projects	GET	Retrieve list of projects
/db/{database}/projects/{project_id}	GET	Retrieve project information
/db/{database}/projects/{project_id}/isolates	GET	Retrieve list of isolates belonging to a project
/db/{database}/submissions	GET	Retrieve list of your submissions
/db/{database}/submissions	POST	Create new submission
/db/{database}/submissions/{submission_id}	GET	Retrieve submission record
/db/{database}/submissions/{submission_id}	DELETE	Delete submission record
/db/{database}/submissions/{submission_id}/messages	GET	Retrieve submission correspondence
/db/{database}/submissions/{submission_id}/messages	POST	Add submission correspondence
/db/{database}/submissions/{submission_id}/files	GET	Retrieve list of supporting files uploaded for submission
/db/{database}/submissions/{submission_id}/files	POST	Upload submission supporting file
/db/{database}/submissions/{submission_id}/files/{filename}	GET	Download submission supporting file
/db/{database}/submissions/{submission_id}/files/{filename}	DELETE	Delete submission supporting file

* Substitute field values where terms are enclosed in {curly brackets}. Submission methods require OAuth authentication to identify the user.

**Figure 1.**
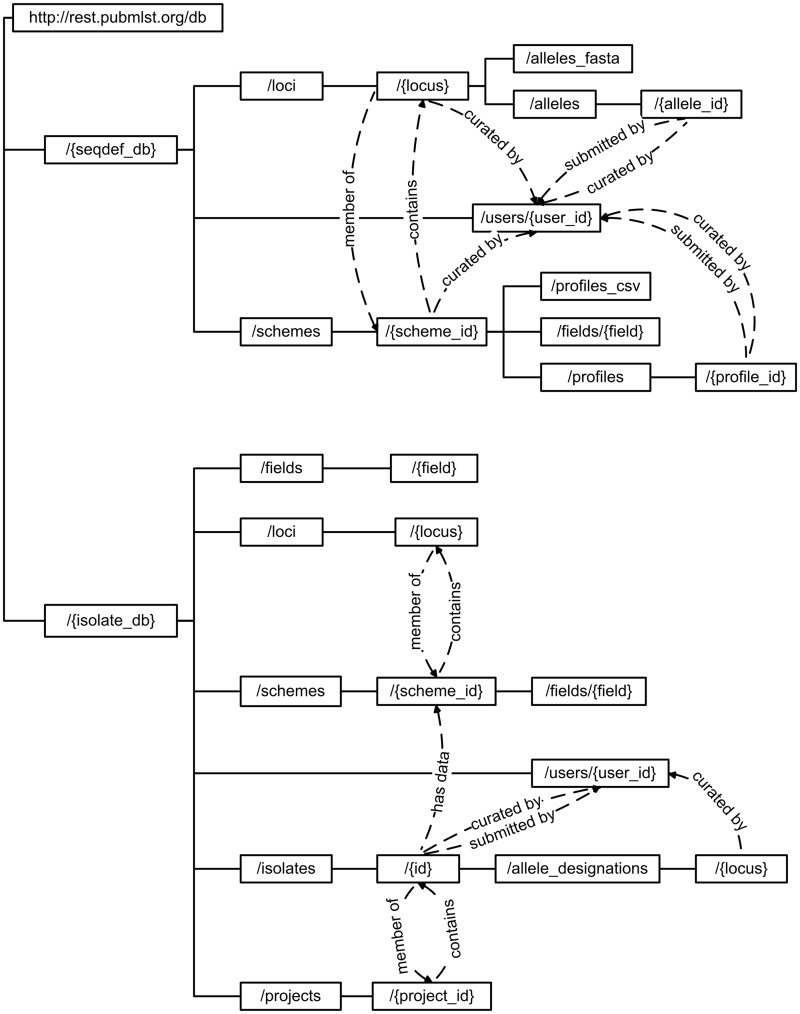
Schematic of API structure. Method calls are written within boxes, with the complete URL constructed by appending the hierarchical values from the root. Terms written in curly brackets represent specific entity values. Dashed lines show where the output from one method include calls to further methods. For example, a scheme record will link to member loci, whereas a locus record will include links to schemes of which it is a member.

Most available methods are discoverable from the root entry point (http://rest.pubmlst.org/), which lists the database resources available. Following universal resource identifiers (URIs) returned from any of these database resources lists further URIs leading to hierarchies of data that can be explored (Figure 1).

**Figure 2.**
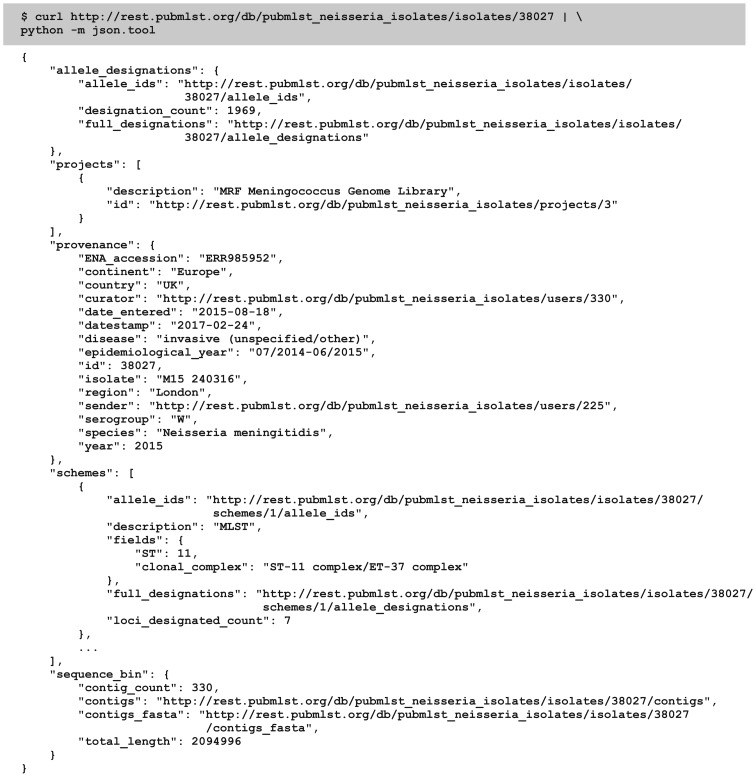
Retrieving an isolate record. A HTTP GET call to http://rest.pubmlst.org/db/{database}/isolates/{isolate_id} can be used to return an isolate record (highlighted – database configuration name and isolate id number are substituted for the variables). The abbreviated response (not all scheme data is shown) is piped through the Python json.tool to format it for readability.

Extracting all the information stored about entities is possible by following the multiple returned URIs. For instance, an isolate record (Figure 2) will contain:

- URIs to the user records of the sender and curator, providing user affiliation and curator contact information;
- URIs describing the assembled contigs, each of which will have information concerning how it was generated, by who and when;
- URIs to allele designations for particular typing schemes and their contig positions;
- A list of PubMed ids of publications including the isolate;
- URIs to projects that the isolate record is a member of.

While this detailed information is available, often a user may only be interested in retrieving known alleles and allelic profiles, or the complete genome assembly for an isolate, without the complete associated metadata, and these can be downloaded in bulk formats such as FASTA or tab-delimited text with a single method call.

## Searching data

**Figure 3.**
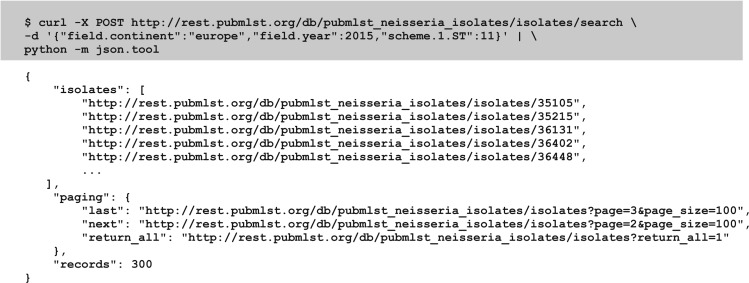
Constructing a query to an isolate database. A query of the isolate database can be performed using a HTTP POST call with the search parameters formatted as JSON. Here we query the *Neisseria* database for all ST-11 isolates, sampled in 2015 from Europe. The curl command line tool can be used to send this query (highlighted). The abbreviated response (only five isolate records are shown rather than the default 100) is piped through the Python json.tool to format it for readability.

Most method calls are for returning specific data records or all records of a particular type. While this is satisfactory for synchronizing molecular typing sequence definitions needed for local use, more advanced searches are also possible. Search parameters can be combined and JSON-encoded in a POST call to query the isolate database, returning a list of URIs to isolate records (Figure 3). Parameters that can currently be included are:

- Provenance metadata fields, for example country, year, or serogroup, although these may vary depending on which database is being queried;
- Allelic designations;
- Typing scheme fields such as sequence type (ST) or clonal complex.

It is also possible to query an allelic sequence with a POST call to the sequence definition database to look up its allele designation. Additionally, there are methods that return descriptions of available provenance fields and the loci and fields that make up typing schemes, making it feasible to replicate selected data structures.

## Paging and filtering

Some method calls are likely to return large amounts of data, so these return paged results along with an indication of the total set size to ensure that the server, client and network are not overwhelmed, especially as the user or client software may sometimes only require the total number of results. The default page size is 100 records. If there are more records than this in the returned dataset, the JSON response will include a paging object that contains URIs to the next, previous, first and/or last pages. It also includes a URI that will return the complete set of data in a single page. The current page and page size can be modified by appending parameters to the URI, e.g. http://rest.pubmlst.org/db/pubmlst_neisseria_isolates/isolates?page=2&page_size=10.

The API also supports paging using request headers. If either of the headers X-OFFSET or X-PER-PAGE are included in the request, this overrides values passed as arguments in the URI and disables inclusion of the paging object in the JSON response. The response headers include X-OFFSET, X-PER-PAGE and X-TOTAL-PAGES for all methods supporting paging.

Some calls can be filtered further to return results that were added after a particular date, or updated after a particular date. This is done by appending the ‘added_after’ or ‘updated_after’ parameter with the value set to the required date in ISO 8601 format (yyyy-mm-dd), e.g. http://rest.pubmlst.org/db/pubmlst_neisseria_seqdef/loci/abcZ/alleles_fasta?added_after=2017-02-28.

## Authentication

While most of the data on PubMLST is publicly available without registration, there are circumstances where a user may need to authenticate themselves, such as to submit data for curation. Access to specific data resources can also be limited to registered users or project members. To facilitate this via the API, OAuth authentication (version 1.0 A) methods are supported. These allow users to delegate access to their PubMLST account to third-party tools or local scripts without the need to share credentials. The workflow for OAuth authentication is as follows:

- The third-party software developer requests a consumer key and consumer secret specific to their application;
- The application gets a request token via an API call and directs the user to an authorization page on PubMLST where they log in by entering their credentials;
- If the entered user credentials match and are registered for a specific resource, a single use verifier code is provided. This code is valid for 1 h;
- The third-party application uses the request token and verifier code to make a signed request for an access token and secret. This access token is valid indefinitely but can be revoked by both the user and the PubMLST administrators;
- The third-party application uses the access token to make a signed request for a session token. This session token is valid for 12 h;
- All calls to protected resources are signed using the session token, consumer token and their respective secrets.

Once the user has delegated access to their account and an access token issued, all further handshakes required to obtain session tokens can be automated, facilitating unattended server-to-server interaction.

## Data submissions

Most submissions for curation are currently received via a web-based submission system that performs basic checks for data correctness before accepting a submission and notifying the appropriate curators. Assignment of some record types require the inclusion of supporting data, such as trace files for new sequences determined by Sanger sequencing and messaging to the teams of curators who handle the submissions. The API integrates with this system with method calls for creating new submissions, adding correspondence, and uploading, downloading and deleting supporting files. Submitting users need to be registered for the database and submission method calls are signed and authenticated so that they are identified on the system. This will allow third-party applications to automatically submit data on behalf of a user, so that new assignments can be seamlessly integrated in to an existing workflow or for data submission to be built in to a data generation pipeline.

## Sample scripts and worked examples

A collection of sample scripts for interacting with the API can be found at https://github.com/kjolley/BIGSdb/tree/develop/scripts/rest_examples. There are versions written in both Perl and Python to demonstrate interaction of the API from both languages. These include test clients that handle the OAuth authentication for accessing restricted resources and for submitting data to the curation queues. A test database has been setup so that dummy submissions can be made.

## Discussion

The RESTful API makes the large amount of curated, structured data on PubMLST accessible for programmatic access, substantially increasing the value of the resource by facilitating data integration with localized analysis tools and pipelines. The concepts and semantic relationships defined for sequence-based typing methods have been defined in the TypOn ontology (13) and the parts of the RESTful API that involve these entities can be mapped on to this ontology. The common use case for the API would be to synchronize local molecular typing databases to ensure that these can be kept up to date with defined nomenclatures. The wide range of methods available, however, also enables more advanced exploitation of data such as retrieving genome assemblies based on specified criteria for automated importation for local analysis or visualization. The data submission methods can be leveraged by third party bioinformatics tools to streamline the process of obtaining new sequence variant designations by sending data to the curator queue and handling the response, removing the need for their end users to manually submit data for assignment.

The API can be expanded with further functionality as needs arise. One potential avenue for future development is to allow curation directly via the API, facilitating direct data upload and editing outside of the standard curation web interface. This would neccessarily be restricted to authenticated curators who would be able to delegate access to validated curation tools, automating the process as much as possible. Other possibilities emerge if some of the BIGSdb analysis methods can be made available from the API, for example the comparative genomics analysis implemented in Genome Comparator or tree drawing using the PhyloTree plugin. Initiation of these kinds of analyses directly may facilitate alternative interfaces such as dashboards focused towards particular tasks and categories of user.
